# Semi-automatic spray pyrolysis deposition of thin, transparent, titania films as blocking layers for dye-sensitized and perovskite solar cells

**DOI:** 10.3762/bjnano.9.105

**Published:** 2018-04-10

**Authors:** Hana Krýsová, Josef Krýsa, Ladislav Kavan

**Affiliations:** 1 J. Heyrovský Institute of Physical Chemistry of the CAS, v. v. i., Dolejškova 2155/3, 182 23 Prague 8, Czech Republic,; 2 University of Chemistry and Technology Prague, Technická 5, 166 28 Prague 6, Czech Republic

**Keywords:** blocking films, FTO, solar cells, spray pyrolysis deposition, titanium dioxide

## Abstract

For proper function of the negative electrode of dye-sensitized and perovskite solar cells, the deposition of a nonporous blocking film is required on the surface of F-doped SnO_2_ (FTO) glass substrates. Such a blocking film can minimise undesirable parasitic processes, for example, the back reaction of photoinjected electrons with the oxidized form of the redox mediator or with the hole-transporting medium can be avoided. In the present work, thin, transparent, blocking TiO_2_ films are prepared by semi-automatic spray pyrolysis of precursors consisting of titanium diisopropoxide bis(acetylacetonate) as the main component. The variation in the layer thickness of the sprayed films is achieved by varying the number of spray cycles. The parameters investigated in this work were deposition temperature (150, 300 and 450 °C), number of spray cycles (20–200), precursor composition (with/without deliberately added acetylacetone), concentration (0.05 and 0.2 M) and subsequent post-calcination at 500 °C. The photo-electrochemical properties were evaluated in aqueous electrolyte solution under UV irradiation. The blocking properties were tested by cyclic voltammetry with a model redox probe with a simple one-electron-transfer reaction. Semi-automatic spraying resulted in the formation of transparent, homogeneous, TiO_2_ films, and the technique allows for easy upscaling to large electrode areas. The deposition temperature of 450 °C was necessary for the fabrication of highly photoactive TiO_2_ films. The blocking properties of the as-deposited TiO_2_ films (at 450 °C) were impaired by post-calcination at 500 °C, but this problem could be addressed by increasing the number of spray cycles. The modification of the precursor by adding acetylacetone resulted in the fabrication of TiO_2_ films exhibiting perfect blocking properties that were not influenced by post-calcination. These results will surely find use in the fabrication of large-scale dye-sensitized and perovskite solar cells.

## Introduction

Dye-sensitized solar cells (DSSCs), solid state dye-sensitized solar cells (SSDSSCs) and perovskite solar cells (PSCs) are attractive alternatives to solid state photovoltaics at competitive cost. The general concept of a DSSC is based on a liquid junction photo-electrochemical cell with a nanocrystalline TiO_2_ photoanode that is sensitized with a dye. This is in contact with an electrolyte solution with a redox mediator which transports holes from the photo-oxidized dye towards the counter electrode. In SSDSSCs or PSCs, the photogenerated holes are transported by a solid conductive material (e.g., spiro-OMeTAD) [1–2]. This is accompanied by the undesirable back reaction of photoinjected electrons with the hole-transporting medium or the oxidized mediator. This reaction occurs both at the TiO_2_ surface and at the exposed areas of the F-doped SnO_2_ (FTO) conducting glass that are not covered by the titanium dioxide nanoparticles. In liquid-type DSSCs there is a relatively small recombination current as compared to FTO, which becomes critical in SSDSSCs and perovskite solar cells [3].

For proper function of these solar cells, a semiconducting nonporous blocking layer of oxide (usually TiO_2_ or SnO_2_) must be deposited on top of FTO to prevent recombination on this surface [3–5]. Blocking layers (BLs) can be fabricated by spray pyrolysis [3,6], magnetron sputtering [7], electrochemical deposition [8] spin coating [9–10], dip coating [11] and atomic layer deposition (ALD) [3].

From the viewpoint of low-cost processing and easy upscaling, spray pyrolysis deposition (SPD) is the most practical option for the fabrication of solar cells. 120–200 nm thick, compact, TiO_2_ layers were found to be optimal for SSDSSCs [12]. The same layers (with optimized thickness of 30–35 nm) turned out to be useful for PSCs, where they clearly outperformed the layers made by spin coating [9]. Matteocci et al. [13] used a modified precursor solution which was obtained by addition of acetylacetone to the conventional formulation (i.e., titanium diisopropoxide bis(acetylacetonate)) in ethanol. They fabricated compact TiO_2_ blocking layers (via SPD) for poly(3-hexylthiophene) SSDSSCs. Their optimised BLs showed an overall increase in the solar cell efficiency, but the blocking function was not quantified, for example, by analysis of the pinhole area [13]. This was one of the motivations for this work.

The blocking properties are often significantly impaired by calcination at 500 °C (which is mandatory for the subsequent fabrication of mesoporous TiO_2_ thick films). Even though SPD is a popular technique for the fabrication of BLs and the resulting TiO_2_ films are less sensitive to calcination, the blocking properties are inferior to those with films prepared by electrodeposition or ALD for example [3,8]. Therefore the central motivation for this work was SPD fabrication of blocking TiO_2_ films using conventional [6] and novel [13] spray protocols using a semi-automatic spray device, enabling reproducible and uniform thin transparent titania films to be achieved with good blocking properties and low sensitivity to calcination at the same time. Such fabricated BL TiO_2_ films were characterized by photo-electrochemical measurements in aqueous electrolyte solution. The blocking ability was quantified by cyclic voltammetry with a K_3_[Fe(CN)_6_]/K_4_[Fe(CN)_6_] model redox probe [3].

## Experimental

Two types of FTO glass supports were used with the same nominal sheet resistivity (as declared by the supplier, 8 Ω/sq) and thickness (2.2 mm): TEC 8 from Libbey-Owens-Ford, labelled as FTO(A) and TEC 8 from Dyesol labelled as FTO(B). Our own measurement of the sheet resistivity showed 12.5 Ω/sq and 7.9 Ω/sq for FTO(A) and FTO(B), respectively. FTO glass slides were cleaned in ethanol and acetone. TiO_2_ layers were deposited by semi-automatic spray coating set up using two different precursors (i) an ethanolic solution of titanium diisopropoxide bis(acetylacetonate) (abbreviated TAA [6], at concentrations of 0.05 M and 0.2 M) and (ii) an ethanolic solution of titanium diisopropoxide bis(acetylacetonate) with added acetylacetone with total molar ratio Ti/acetylacetone 1:2.4 (abbreviated TAA-AcAc) [13]. Spraying was carried out by semi-automatic spray device consisting of a nozzle (EST 616, Czech Republic) fed with compressed air (3 bar) and a solution of precursor (flux of 2.7 cm^3^/min). The nozzle was mounted on a linear stage (velocity 20 cm/s) and the precursor solution was delivered downwards to a heated support (150, 300 or 450 °C). The working area of deposition was up to 20 × 30 cm, whereby upscaling is technically feasible. During each spray process (lasting approximately 0.5 s) a significant decrease of the temperature of the heating plate occurred for several seconds. To avoid this undesired cooling, the next spraying was performed at regular time intervals (30 s) which were necessary for complete layer formation and restoration of the original substrate temperature. This technique allows for the fabrication of films using various numbers of spray cycles (SCs). During the coating procedure, a narrow area at the edge of the FTO glass substrate was protected against deposition of the TiO_2_ by a mask. This allowed for an ohmic contact to the electrode for subsequent electrochemical and photo-electrochemical tests. Table 1 gives a summary of the fabricated samples. The variable growth parameters were: FTO type (A, B), precursor concentration, deposition temperature, number of spray cycles and influence of the post-calcination step (in air at 500 °C for 1 h).

**Table 1:** Overview of the experimental conditions for spray deposition of titania blocking layers deposited on top of FTO conducting glass.

Precursor type and concentration	FTO glass	Substrate temperature/ °C	Number of spray cycles

0.05 M TAA	A	150	100
	A	450	100
0.05 M TAA	A,B	450	50
	A,B	450	100
	A,B	450	200

0.2 M TAA	B	450	20
	B	450	30
	B	450	40
	B	450	50
	B	450	60
	B	450	70

0.2 M TAA- AcAc	B	450	20
	B	450	30
	B	450	40
	B	450	50
	B	450	60
	B	450	70

The produced titania films were characterized by X-ray diffraction (XRD) (PANanalytical X’Pert PRO, Cu Kα radiation, 1D XCelerator detector, PANanalytical B.V., Almelo, Netherlands), scanning electron microscopy (SEM) (Hitachi FE SEM S-4800 microscope), and UV–vis spectroscopy (Varian CARY 100 with integrating sphere DRA-CA-30I and 8° reflectance geometry, Varian, USA). X-ray fluorescence spectroscopy (XRF) analysis was performed on an ARL 9400 XP sequential WD-XRF spectrometer (Thermo ARL, Switzerland) equipped with a Rh anode end window and a 4GN-type X-ray tube fitted with a 50 μm Be window. The obtained data were evaluated by Uniquant 4 software.

Photo-electrochemical and electrochemical experiments were carried out in a one-compartment three-electrode cell using an Autolab Pgstat 101 instrument controlled by the NOVA software. The TiO_2_ thin film on FTO was the working electrode, the reference electrode was Ag/AgCl (3 M KCl) and a platinum rod was used as the counter electrode. The TiO_2_ films were illuminated from the front side by a Hg lamp (Oriel), and the TiO_2_ film area (1 cm^2^) was defined by a Teflon tape mask. Optical filters were used to select the wavelength range of 320–380 nm. Intermittent light was applied at 10 s dark/light intervals and the working electrode potential was swept with a scan rate of 5 mV s^–1^. 0.1 M Na_2_SO_4_ (pH 10) was used as the electrolyte solution for photo-electrochemical experiments [14]. The blocking properties of the deposited layers were evaluated by cyclic voltammetry (CV) in aqueous electrolyte solution composed of 0.5 mM K_4_[Fe(CN)_6_], 0.5 mM K_3_[Fe(CN)_6_] in 0.5 M KCl, pH 2.5.

## Results and Discussion

**Figure 1:**
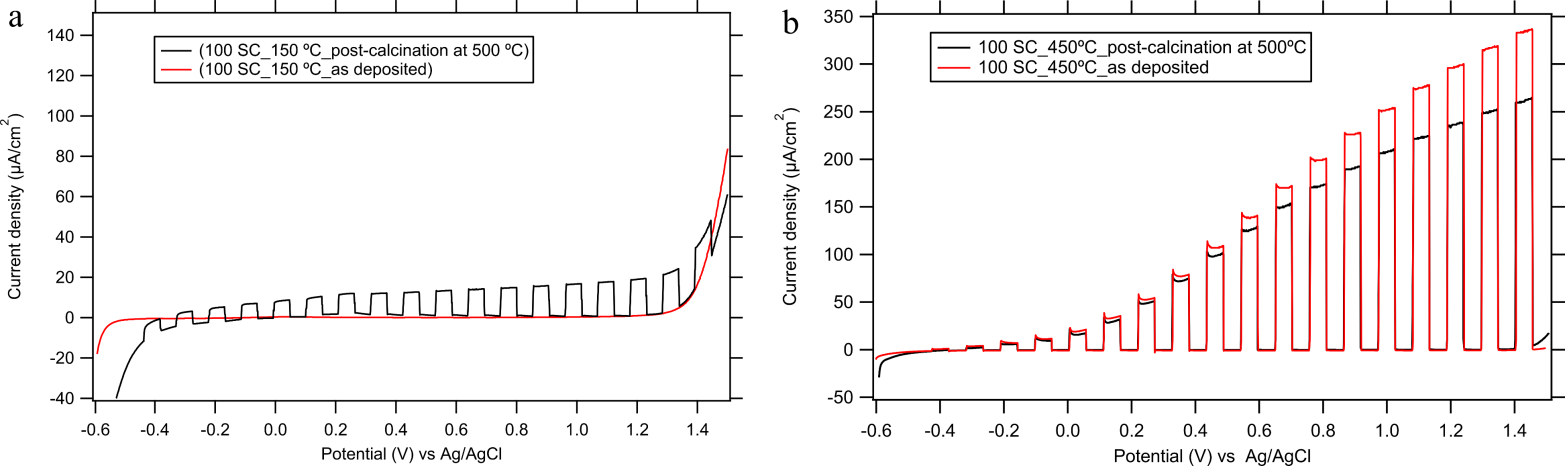
Linear sweep voltammetry under intermittent UV light excitation (Hg lamp 320–380 nm) of intensity 7.5 mW/cm^2^. Comparison of TiO_2_ films (100 SCs) deposited on FTO (A) at 150 °C (a) and 450 °C (b) with and without subsequent calcination. The electrolyte solution was 0.1 M Na_2_SO_4_ (pH 10), dark/light intervals of 10 s were applied.

To optimize the SPD conditions, we initially tested the effect of deposition temperature. Our TiO_2_ films were grown using 100 spray cycles (SCs) using a TAA solution of concentration 0.05 M, and the temperature of the FTO substrates was held at 150, 300 and 450 °C, respectively. Figure 1a shows the UV-photo-electrochemical scan of a TiO_2_ film deposited at 150 °C. Without post-calcination at 500 °C, the photocurrent density is negligible. The photoresponse was similarly negligible for the deposition temperature of 300 °C (data not shown). Only after calcination at 500 °C will the low-temperature-grown films become photoactive, where the photocurrent onset was observed at around −0.35 V (Figure 1a). In the applied potential range of 0.25–1.2 V there is a photocurrent plateau at ≈14 μA/cm^2^ at the UV illumination intensity applied (7.5 mW/cm^2^).

For the deposition temperature of 450 °C, our titania films are photoactive already in the as-grown state (see Figure 1b). These TiO_2_ films exhibit the photocurrent onset at around –0.3 V but there is no plateau at larger potentials. Instead, the photocurrent increases monotonically with the applied potential. The effect of post-calcination is opposite compared to the low-temperature films deposited at 150 °C (cf. Figure 1a). For instance, at the potential of 1.2 V, we observe a photocurrent density of ≈300 μA/cm^2^ for the as-grown film (at 450 °C) but only ≈240 μA/cm^2^ for the same film, but post-calcined at 500 °C.

The onset of anodic dark current for a TiO_2_ film deposited at 450 °C occurs at a potential of ≈1.45 V. On the other hand the onset potential of the dark anodic current is downshifted to ≈1.3 V for TiO_2_ films deposited at 150 °C. A similar dark current onset potential (of ≈1.3 V) was observed on bare FTO under comparable experimental conditions [15–16]. This suggests that the TiO_2_ films deposited at 150 °C and post-calcined at 500 °C contain pinholes and cracks through which the electrolyte can reach the FTO surface. As TiO_2_ is under strong depletion at these potentials, no anodic current of, for example, water oxidation, is expected to flow at the perfectly compact titania electrode in dark.

The blocking quality of our layers was tested in the pH-independent aqueous model redox system K_3_[Fe(CN)_6_]/K_4_[Fe(CN)_6_] [3]. Nernstian pH-dependence is demonstrated by the flat-band potential, φ_FB_, of a single-crystal anatase electrode (Equation 1) [17]:

[1]
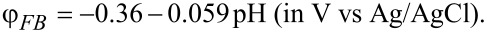


As compared to this TiO_2_ (anatase) flat-band potential, the redox potential of [Fe(CN)]_6_^3−^/[Fe(CN)]_6_^4−^ (0.24 V vs Ag/AgCl) is sufficiently positive in aqueous electrolyte solutions at all pH values. As a result, a rectifying interface, at which no anodic current of [Fe(CN)_6_]^4−^ oxidation flows, is obtained with high-quality titania (anatase) blocking layers because at these potentials titania is in the depletion regime. The cathodic current onset (beginning [Fe(CN)_6_]^3−^ reduction) appears at potentials negative to the φ_FB_ when the titania electrode is in the accumulation regime, mimicking metallic behaviour. Defects, such as cracks in the blocking layer are thus sensitively identified by the occurrence of anodic voltammetric current assigned to the [Fe(CN)_6_]^4−^ oxidation at the exposed FTO areas in pinholes [3].

**Figure 2:**
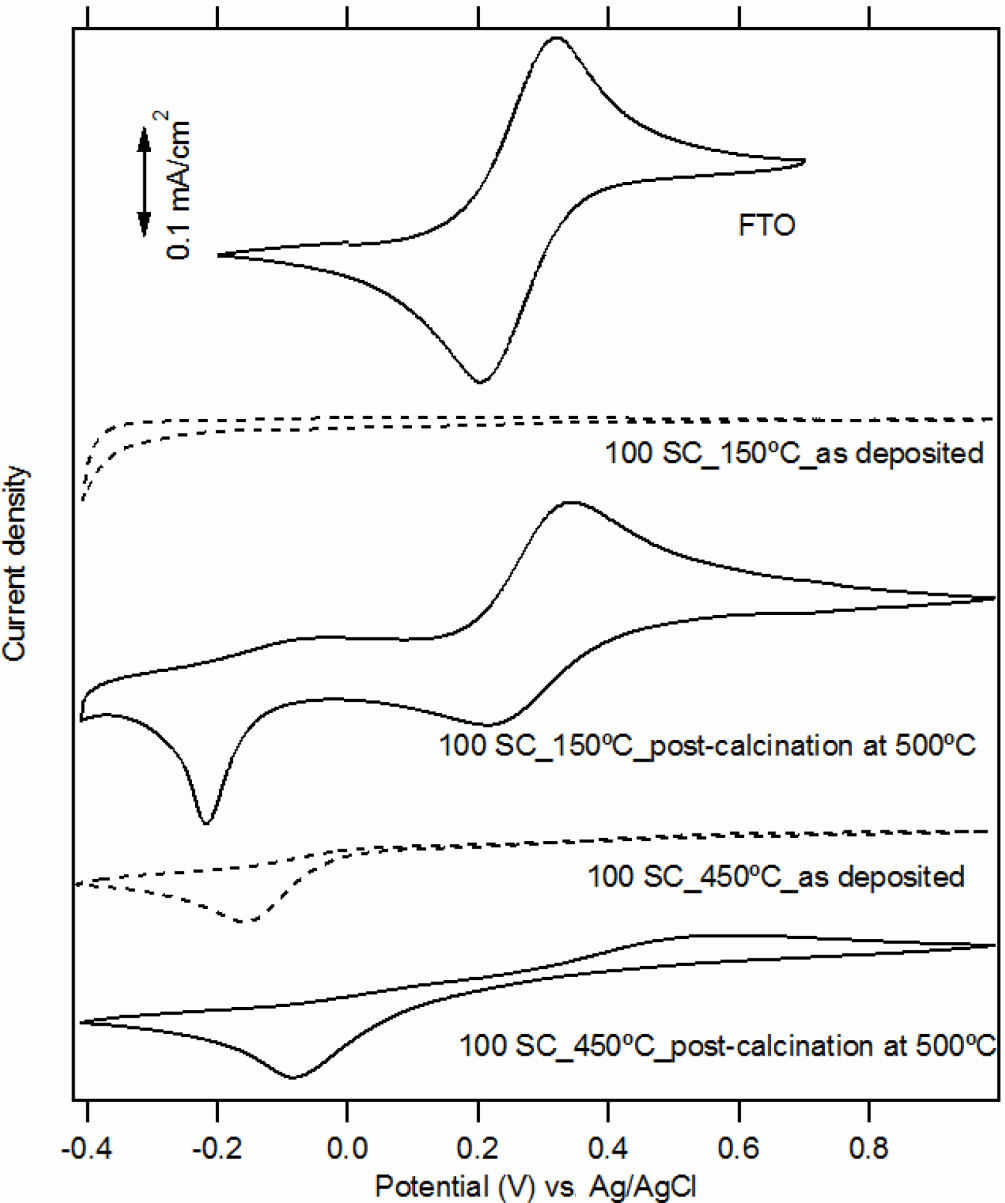
Cyclic voltammograms on an uncoated FTO(A) electrode and one covered by TiO_2_ films consisting of 100 SCs of TAA (0.05 M). Scan rate 50 mV/s. Electrolyte solution was 0.5 mM Fe(CN)_6_^3−/4−^ in aqueous 0.5 M KCl, pH 2.5. The voltammograms (except for FTO) are offset for clarity but the current density scale is identical for all plots.

Figure 2 shows cyclic voltammograms of Fe(CN)_6_^3−/4−^ redox couple at a bare FTO(A) substrate and that for a sample coated with TiO_2_ films (100 SCs) of different deposition. Our TiO_2_ film deposited at 150 °C without post-calcination displays perfect blocking properties, although it is photo-electrochemically inactive (cf. Figure 1a). Post-calcination of this film causes severe deterioration of its blocking function. The cyclic voltammogram Fe(CN)_6_^3−/4−^ is similar to that of pure FTO. The presence of TiO_2_ manifests itself solely by a cathodic peak at ≈−0.2 V which is assigned to the reduction of ferricyanide at the TiO_2_ surface [3]. On the other hand, the as-grown TiO_2_ film deposited at 450 °C has perfect barrier properties, which are less perturbed by the post-calcination.

**Figure 3:**
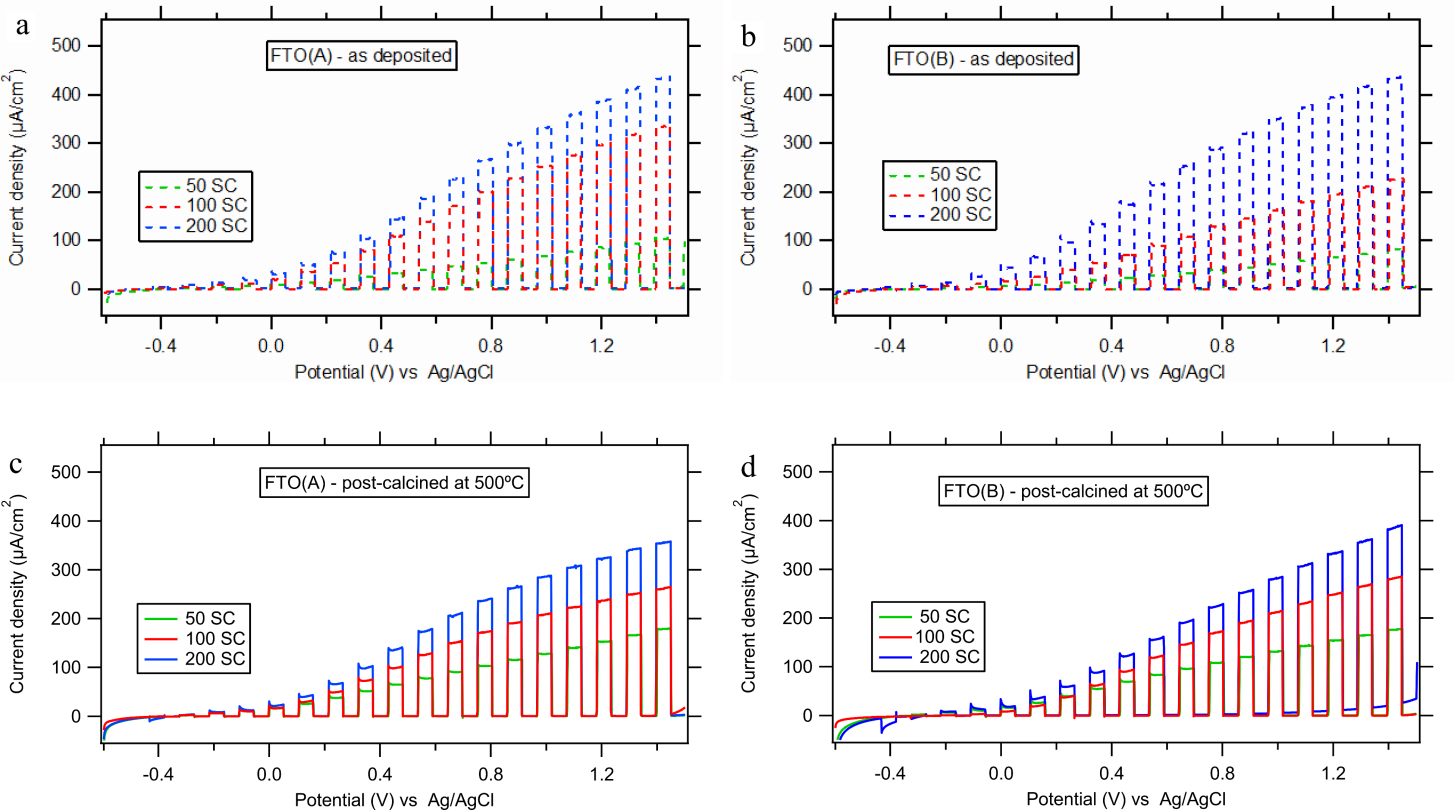
Linear sweep voltammetry under intermittent UV light (Hg lamp 320–380 nm; intensity 7.5 mW/cm^2^). Precursor: 0.05M TAA, deposition temperature: 450 °C, electrolyte solution: 0.1 M Na_2_SO_4_ (pH 10), dark/light interval: 10 s. (a,c) Films deposited on FTO(A) 50, 100 and 200 SCs. (b,d) Films deposited on FTO(B) 50, 100 and 200 SCs.

As a next step, we investigated in detail the influence of layer thickness, which was adjusted by the number of spray cycles (SC, 50–200) of 0.05 M TAA solution. We have chosen the deposition temperature of 450 °C and have also investigated the influence of the respective FTO glass substrates (A or B, see Experimental section). Figure 3 shows that the photocurrent density increases with the number of SCs for both the FTO substrates.

The onset potential of the dark anodic current (≥1.4 V) suggests a rather compact character of our films deposited at 450 °C. The onset potential of photocurrent is very similar (around −0.3 V) on both our FTO supports and for various layer thicknesses. There is very small slope of the photocurrent/potential curve around the photocurrent onset and then an almost linear increase of the photocurrent. A similar shape in the polarization curve was observed previously for rutile TiO_2_ films [18]. However, SPD titania films are likely to be of anatase structure as shown by our XRD analysis (see Figure S1 and Figure S2 in Supporting Information File 1) and confirmed by others [3,6,13].

Figure 3a,b shows that the type of FTO substrate used significantly influences the photocurrent on the as-deposited TiO_2_ films. On the FTO(A) substrate, there is a strong increase of photocurrent between 50 and 100 SCs and a less-pronounced increase between 100 and 200 SCs. On the other hand, for FTO(B), the photocurrent increases proportionally with the increase of the number of spray cycles (between 50 and 200 SCs). This FTO-specific behaviour greatly diminishes after post calcination at 500 °C (Figure 3c,d), which results in a very similar influence of the number of SCs on the photocurrent for both the FTO supports.

**Figure 4:**
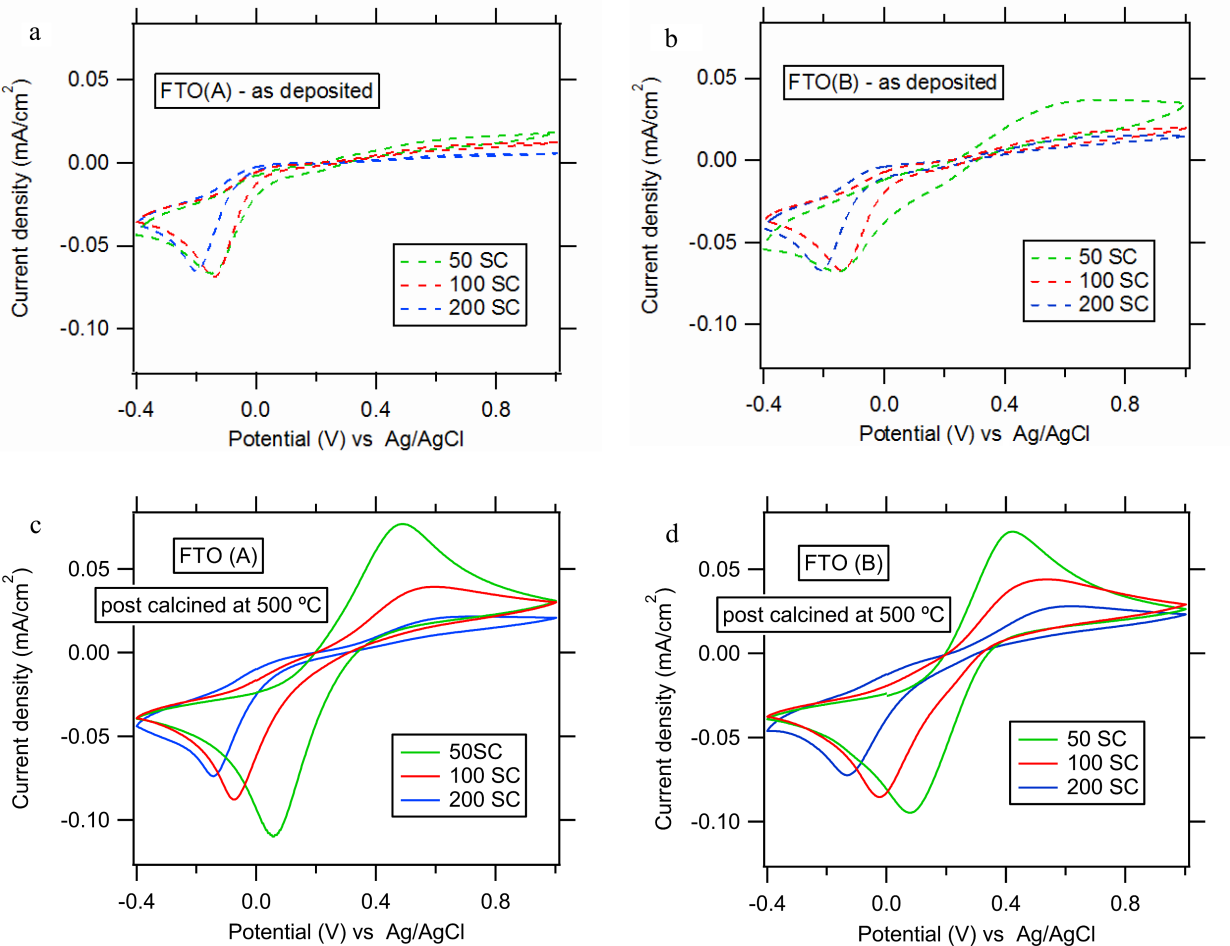
Cyclic voltammograms for TiO_2_ films; precursor: 0.05M TAA, deposition temperature: 450 °C, scan rate: 50 mV/s, electrolyte solution: 0.5 mM Fe(CN)_6_^3−/4−^ in aqueous 0.5 M KCl, pH 2.5. (a,c) films deposited on FTO(A) 50, 100 and 200 SCs, (b,d) films deposited on FTO(B) 50, 100 and 200 SCs.

Cyclic voltammograms of Fe(CN)_6_^3−/4−^ redox couple on pure FTO substrates (A or B) are almost identical (cf. Figure 2 for FTO(A)). Figure 4a shows the corresponding cyclic voltammograms on TiO_2_ films deposited at 450° on FTO(A) substrates (50–200 SCs). All TiO_2_ films have good blocking properties; there is a slight improvement with an increase of the number of SCs, i.e., with the layer thickness. However, after post-calcination, there is a significant deterioration of the blocking properties, and the positive influence of increased SCs on the blocking properties is more apparent (see Figure 4c).

A similar behaviour was observed for the TiO_2_ films deposited on the FTO(B) substrate, but the blocking properties were not as good as than those for TiO_2_ films deposited on FTO(A), especially for the lowest thickness (50 SCs), see Figure 4b. Also, the post calcination at 500 °C results in a significant deterioration of the blocking properties, see Figure 4d.

Although the used substrates (FTO(A) and FTO(B)) are nominally identical (TEC 8; differing solely by the supplier, see Experimental section) we observed a surprisingly strong influence of the substrate type on the blocking properties of titania films which were sprayed over them. More specifically, FTO(A) provides considerably better blocking properties of the corresponding titania films. To address this peculiarity, we investigated these substrates in more detail and the results are summarized below.

**Figure 5:**
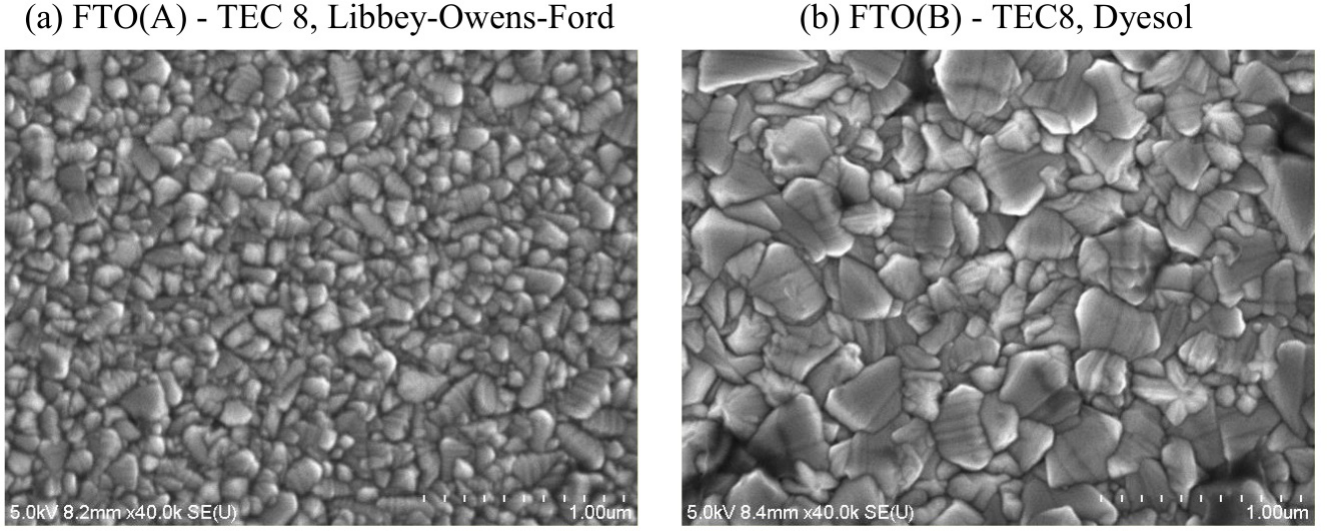
Top view SEM images of FTO glass substrates, (a) FTO(A) and (b) FTO(B).

Figure 5 shows the top surface morphology of both our FTO substrates. The grain size is between 100 and 200 nm for FTO(A), but between 300 and 500 nm for FTO(B). Furthermore, FTO(B) is less transparent than FTO(A), whereby the optical transmittance in the range of wavelengths from 350 to 550 nm is by about 15–20% lower, which is consistent with the difference in the structure. The coherent domain size calculated from X-ray line broadening by the Scherrer equation was 24 nm for FTO(A) and 36 nm for FTO(B). These values are consistent with the difference in the grain size for both the FTO samples. Our own measurement of sheet resistivity provided the following values. FTO(A), TEC8, Libbey-Owens-Ford: 12.5 Ω/sq and FTO (B), TEC 8, Dyesol: 7.9 Ω/sq. This finding is quite surprising and implies that FTO(B) has a higher resistivity (by about 50%) than that declared by the supplier.

The larger grain size of the FTO(B) substrate is the apparent reason for its incomplete coverage by titania film. Figure 4a illustrates this finding, which is particularly expressed for the smallest thicknesses (50 SCs). For a larger number of SCs (100 and 200) the differences between our FTO substrates are damped. The evaluation of the fraction coverage of FTO by the titania blocking layer is based on the voltammetric current density peak, *j*_p_, which follows the Randless−Sevcik equation [3]. The effective pinhole area (EPA) can be expressed by Equation 2 as

[2]
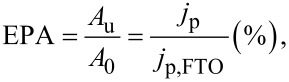


where *j*_p_ is the peak current density (with reference to the projected electrode area, *A*_0_) measured at the actual blocking electrode, *A*_u_ is the uncovered area of FTO and *j*_p,FTO_ is the current density peak measured at a bare FTO electrode. The actual values are listed in Table 2. As suggested in a previous work [3], there are two types of defects in the barrier film: (i) “defect A” – the partially blocked electrode behaves like a “clean” FTO but with a relatively smaller effective area. The relative increase of voltammetric peaks separation (Δ*E*_pp_) on the BL normalized to that for pure FTO is smaller than 3. (Δ*E*_pp_ is defined as the difference between the peak potentials for the Fe(CN)_6_^4−^ oxidation and Fe(CN)_6_^3−^ reduction). (ii) “defect B” – a more complex situation, where the defect causes not only the delamination of the titania film from FTO, but also the slowdown of charge transfer kinetics (accompanied by a strong increase in Δ*E*_pp_).

**Table 2:** Analysis of the effective pinhole area for our as-deposited and post-calcined TiO_2_ films. Data from cyclic voltammetry of Fe(CN)_6_^3−/4−^; scan rate 50 mV/s (experimental conditions same as in Figure 2).

Substrate	Precursor	No. of SCs	*j*_p_ (mA cm^−2^)		EPA (%)		Defect type
			as dep.	calcined	as dep.	calcined	

FTO(A)	–	–	0.15	–	100	–	–
FTO(A)	0.05 TAA	50	0.020	0.075	13	50	A
FTO(A)	0.05 TAA	100	0.013	0.035	9	23	A+B
FTO(A)	0.05 TAA	200	0.006	0.02	4	13	B
FTO(B)	0.05 TAA	50	0.040	0.07	27	47	A
FTO(B)	0.05 TAA	100	0.017	0.03	11	20	A+B
FTO(B)	0.05 TAA	200	0.010	0.015	7	10	B
FTO(B)	0.2 TAA	20	0.010	0.02	7	13	B
FTO(B)	0.2 TAA	50	0.005	0.01	3	7	B
FTO(B)	0.2 TAA	70	0.002	0.005	1	3	B
FTO(B)	0.2 TAA-AcAc	70	0.002	0.002	1	1	B

The barrier properties after post calcination were fairly good for the 0.05 M TAA precursor and the largest layer thickness (200 SCs), but still ≈10–13% of the FTO surface was uncovered by TiO_2_ (Table 2). Therefore, in the next step, the concentration of TAA was increased to 0.2 M to achieve a larger film thickness per one spray cycle. FTO(B) was chosen as a substrate due to the conformity of its measured/declared sheet resistivity and also as a solution for the reduced blocking performance of thin TiO_2_ films deposited on it (cf. Figure 4a,c and Figure 4b,d). The actual TiO_2_ films were deposited by 20 to 70 spray cycles at 450 °C and again the influence of the post-calcination was investigated. The photocurrent response was measured similarly as in Figure 3. For the as-deposited films as well as the calcined films, the photocurrent density slightly increases with increasing number of SCs (from 20 to 40), but remains constant for higher SCs (between 50 and 70). The observed photocurrent density at a potential of 1.2 V was 460 μA/cm^2^ for the calcined TiO_2_ film from 0.2 M TAA (50 SCs). Figure 3b and Figure 3d illustrate that this photocurrent is roughly comparable to that on the calcined thick film from diluted solution (0.05 M-TAA; 200 SCs), i.e., 350 μA/cm^2^.

**Figure 6:**
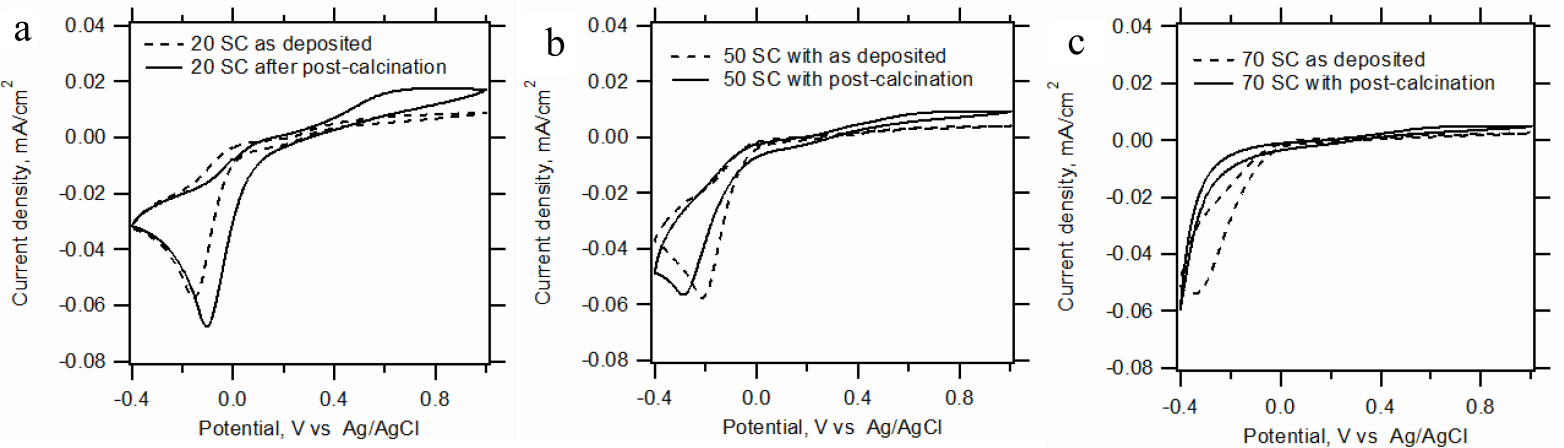
Cyclic voltammograms on a FTO(B) substrate covered by TiO_2_ films of (a) 20 SCs, (b) 50 SCs and (c) 70 SCs of 0.2 M TAA. Scan rate 50 mV/s. Electrolyte solution was 0.5 mM Fe(CN)_6_^3−/4−^ in aqueous 0.5 M KCl, pH 2.5.

Figure S3 in Supporting Information File 1 shows the cyclic voltammograms of FTO(B) covered by TiO_2_ films deposited by 20 SCs of 0.2M TAA. There is a large improvement of the blocking properties when compared to the previous set of samples (Figure 4b,d). More detailed CV data are shown in Figure 6 for the TiO_2_ films consisting of 20, 50 and 70 SCs. In all cases, post-calcination causes a strong deterioration of the blocking properties. The difference between as-deposited TiO_2_ films and those after post-calcination is a dampening with increasing number of SCs. For instance, at 70 SCs, the difference is almost negligible.

**Figure 7:**
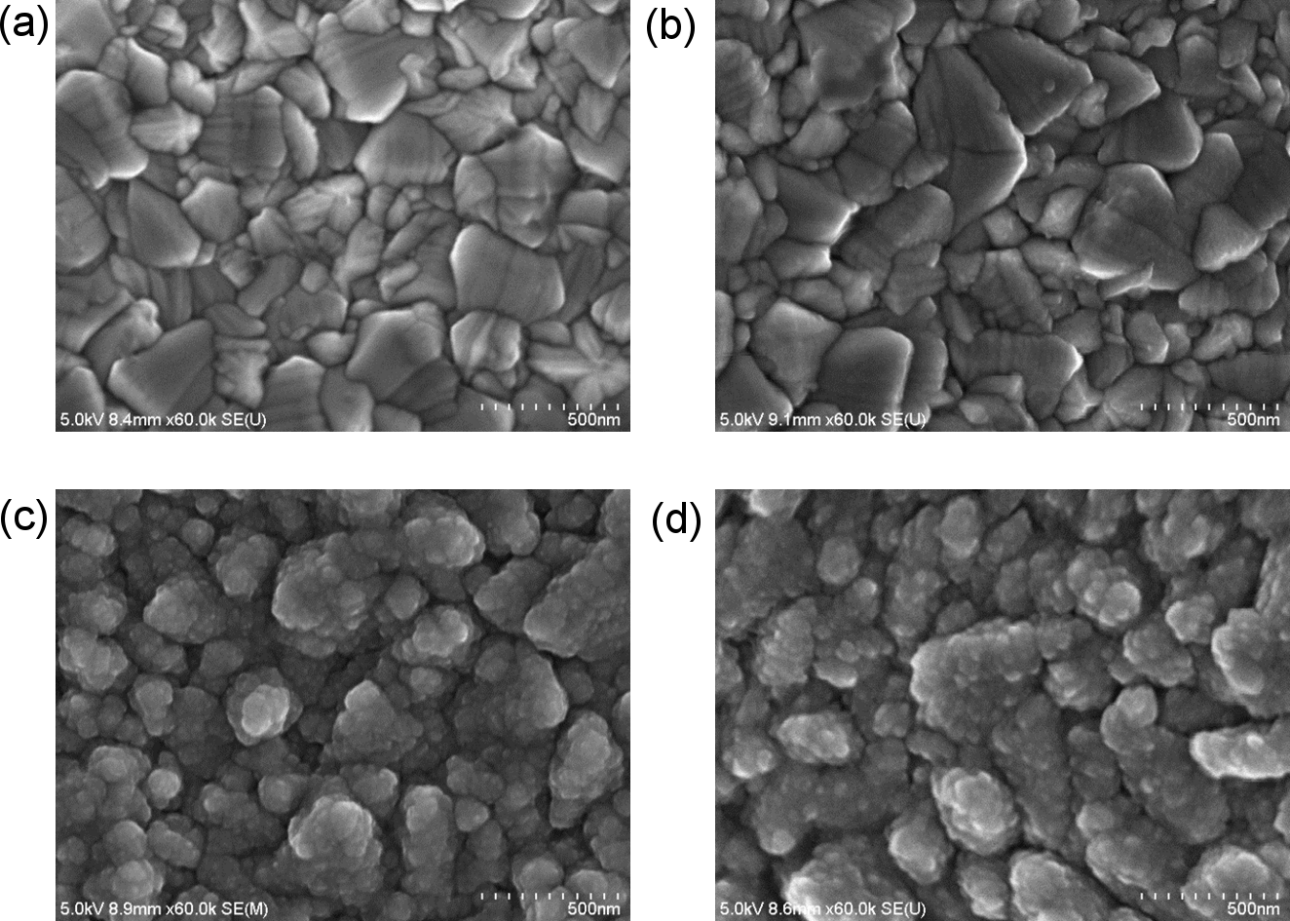
Top view SEM images of a) bare FTO(B), b) FTO(B) covered by 20 SCs of 0.2 M TAA, c) FTO(B) covered by 70 SCs of 0.2 M TAA and d) FTO(B) covered by 70 SCs of 0.2 M TAA with post-calcination at 500 °C.

Figure 7 shows the top surface morphology of our FTO(B) and that of the substrate covered by TiO_2_ films consisting of various SCs. A thin TiO_2_ film (20 SCs) is barely visible on top of FTO (Figure 7b), where the image is dominated by the substrate morphology. With increasing number of SCs, the grains of FTO become covered by small TiO_2_ particles, which provide the blocking function of the electrode (Figure 7c). Interestingly, post-calcination does not result in any significant increase of the TiO_2_ particle size, as seen in Figure 7d.

From the comparison of Figure 6 and Figure 4b,d it follows that the as-deposited and post-calcined TiO_2_ films consisting of 20 SCs (0.2 TAA) have comparable blocking properties to the TiO_2_ film consisting of 200 SCs (0.05 TAA). With increasing number of SCs (from 20 to 70), the negative influence of post-calcination on the blocking properties of TiO_2_ films further diminishes (cf. Figure 6 and the EPA values in Table 2).

**Figure 8:**
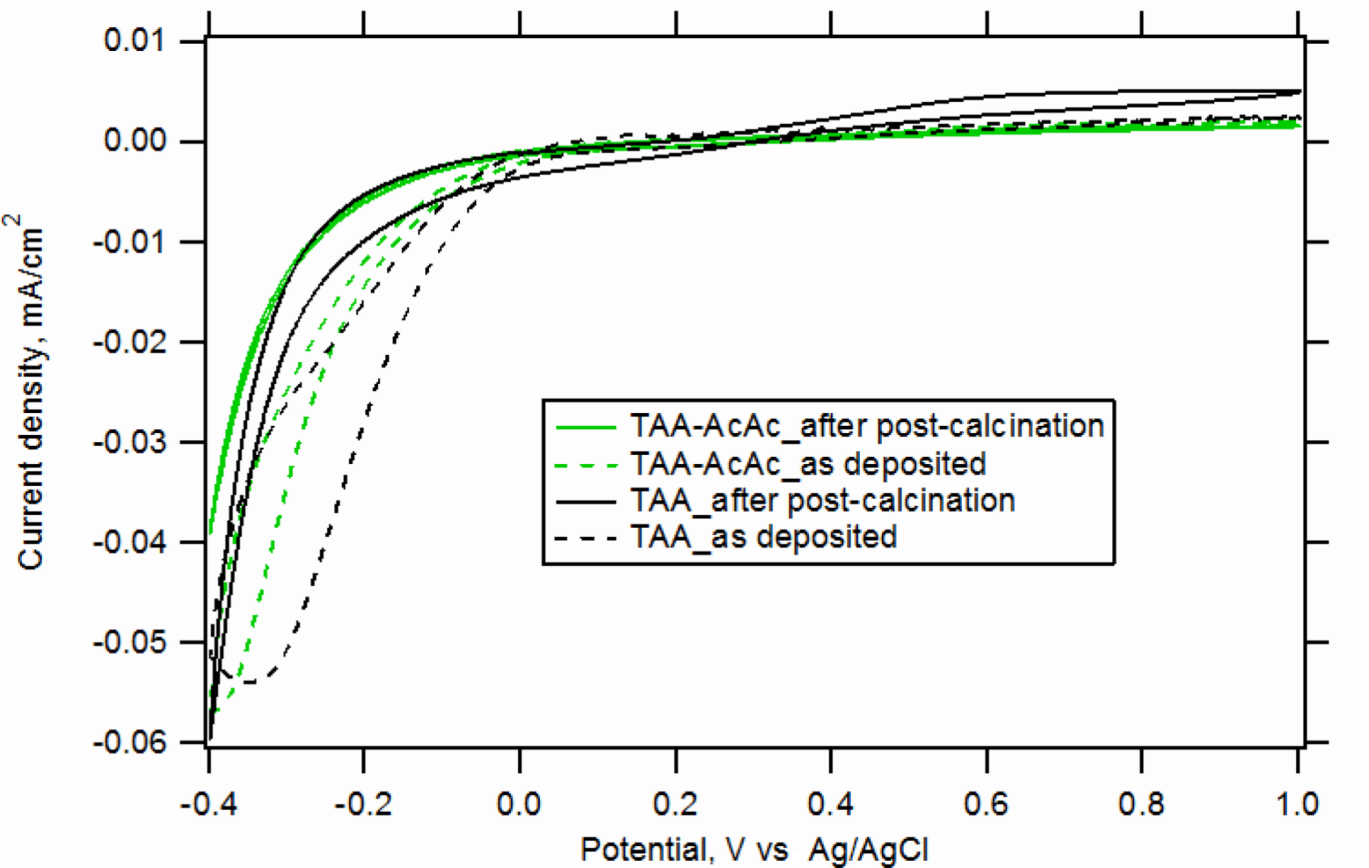
Cyclic voltammograms of FTO(B) covered by sprayed TiO_2_ films made of 70 SCs of 0.2 M TAA and of 0.2 M TAA-AcAc. Scan rate 50 mV/s. Electrolyte solution was 0.5 mM Fe(CN)_6_^3−/4−^ in aqueous 0.5 M KCl, pH 2.5.

In the last step, we investigated the influence of the precursor solution composition. Figure 8 compares the cyclic voltammograms of Fe(CN)_6_^3−/4−^ on FTO(B) covered by TiO_2_ films consisting of 70 SCs by two precursors, 0.2 M TAA and 0.2 M TAA-AcAc, where the latter was enriched with added acetylacetone (see Experimental section). Although the 70 SC TAA film has already quite good blocking properties even after post-calcination, examination of the details in the curves shows that the TAA-AcAc films are still better. The shift of the electroreduction peak, or in some cases the missing electroreduction (at −0.2 V), is a sign of good coverage of the electroactive FTO by the TiO_2_ film, which has properties comparable to those of a perfect anatase single crystal. The latter has a flat band potential (φ_FB_) (cf. Equation 1) at ≈−0.5 V at the given experimental conditions (as in Figure 8). Hence, no electroreduction of ferricyanide to ferrocyanide is expected to occur at −0.2 V, because anatase is in the depletion regime. This is indeed observed for our TAA-AcAc films. Actually, the occurrence of the electroreduction peak at −0.2 V at some other compact (pinhole-free) TiO_2_ layers (cf. Figure 6) is less clear from this point of view. A tentative interpretation could consider the fact that certain TiO_2_ films (e.g., those grown by ALD) do show their φ_FB_ significantly upshifted (by ≈0.3 V), see e.g. [3].

Our conclusion concerning a significant difference in the properties of the BL resulting from the two compositions of spray pyrolysis precursors is a bit empirical, and the comparison of TAA vs TAA-AcAc is not reinforced by deeper discussion of the growth mechanism and structure of the products. Matteocci et al. [13] assumed that acetylacetone can induce functionalization of FTO, aiding in turn an efficient anchoring of TiO_2_ to FTO. This hypothesis is rationalized by the well-known chelating effect of acetylacetone on both organometallic complexes and on oxidic surfaces. The optimization through compositional variation is limited by the growth of complicated species, particularly if the acetylacetone concentration is too high [13]. In a closely related study, Boland et al. [19] used IR spectroscopy to confirm that the precursor solution made by mixing of titanium (IV) isopropoxide with acetylacetone contains an excess of unchelated acetylacetone for the Ti/AcAc ratio ≥3. Hence, we (in accord with the cited works and references therein) assume that our used precursor solution has the optimal composition already.

From the XRD analysis, an anatase crystalline structure was not detected for any as-deposited TiO_2_ films even those consisting of 70 SCs (0.2 M TAA), see Figure S1 in Supporting Information File 1. For post-calcined TiO_2_ films, anatase can be detected only for films consisting of 70 SCs (only one band (θ = 25.4°), see Figure S1 in Supporting Information File 1. Similarly, XRD analysis of the 70 SC TAA-AcAc film shows that the anatase band (θ = 25.4°) is visible only after post-calcination.

Surface XRF analysis of the TiO_2_ film consisting of 50 SCs (0.2 M TAA) shows the content of Ti equal to 8.1 μg/cm^2^. Assuming that the film contains only TiO_2_ we obtain the mass of our TiO_2_ to be 13.3 μg/cm^2^ and the corresponding thickness of the TiO_2_ film (using the density of 3.8 mg/cm^3^) equal to 36 nm. Analogously, the thicknesses of our TiO_2_ films consisting of 20, 50 and 70 SCs (0.2 M TAA) are 14, 36 and 50 nm, respectively. This rather low layer thickness together with the very small size of the sprayed TiO_2_ particles (see Figure 7c,d) is responsible for the very weak XRD response.

We should note that our layer thicknesses are considerably smaller than those reported previously for dye-sensitised solar cells (the thicknesses were typically 100–200 nm) [6,13], and are in the thickness region which was optimized previously for perovskite solar cells (30–35 nm) [9,11]. However, the cited work used solely the stoichiometric titanium diisopropoxide bis(acetylacetonate) without any added acetylacetone. Our results support the earlier finding by Matteocci et al. [13], that the addition of extra acetylacetone for BL fabrication is beneficial for the efficiency of poly(3-hexylthiopene) solar cells. Here we extended this finding by an analysis of the blocking function of these layers. To the best of our knowledge, this analysis as well as the systematic screening of deposition conditions is carried out in this work for the first time.

## Conclusion

Transparent TiO_2_ films were prepared by spray pyrolysis using precursors consisting of an ethanolic solution of titanium diisopropoxide bis(acetylacetonate) (TAA) with or without additional acetylacetone added. The films were deposited on FTO glass substrates at temperatures of 150, 300 and 450 °C using a semi-automatic spray device, enabling control of the film homogeneity and thickness over a large area. The minimal deposition temperature of 450 °C was identified for obtaining the photo-electrochemically active, as-grown films. An increase in the precursor concentration affords the corresponding increase of layer thickness normalized to the number of the deposition cycles. The need of careful selection of an FTO substrate is highlighted by a comparison of two nominally identical products from two suppliers. The as-deposited TiO_2_ films (at 450 °C) of thickness ≈15 nm prepared from precursor TAA exhibit good blocking properties with an effective pinhole area (EPA) smaller than 10%. However, post-calcination of the as-grown films at 500 °C had a negative influence on the blocking properties. The problem could be addressed by increasing the number of spray cycles (thickness ≈50 nm) so that an EPA of ≈3% is achieved. The negative influence of the post-calcination is further minimized by deliberate addition of acetylacetone to the precursor solution. In this way, high-quality barrier films were fabricated (EPA ≈1%) which do not suffer from any loss in blocking function after subsequent post-calcination. To the best of our knowledge, the unperturbed blocking utility of our calcined films is shown here for the first time. Furthermore, good blocking is observed for calcined films, which are similar or thinner than those reported earlier by spray pyrolysis deposition. This finding is significant for the fabrication of DSSCs, SSDSSCs and perovskite solar cells.

## Supporting Information

File 1 Additional experimental data.
