# Supplementary material for: Semi-automatic spray pyrolysis deposition of thin, transparent, titania films as blocking layers for dye-sensitized and perovskite solar cells
Source: Beilstein J Nanotechnol. 2018 Apr 10;9:1135–45. doi: 10.3762/bjnano.9.105 (PMC5905270; doi:10.3762/bjnano.9.105)
Supplement: File 1 — Additional experimental data. [file Beilstein_J_Nanotechnol-09-1135-s001.pdf]

Supporting Information

for

# Semi-automatic spray pyrolysis deposition of thin, transparent, titania films as blocking layers for dye-sensitized and perovskite solar cells

Hana Krýsová[1], Josef Krýsa[2*], and Ladislav Kavan[1]

Address: [1]J. Heyrovský Institute of Physical Chemistry of the CAS, v. v. i., Dolejškova 2155/3, 182 23 Prague 8, Czech Republic, and [2]University of Chemistry and Technology Prague, Technická 5, 166 28 Prague 6, Czech Republic

Email: Josef Krýsa - josef.krysa@vscht.cz

[*] Corresponding author

## Additional Experimental Data

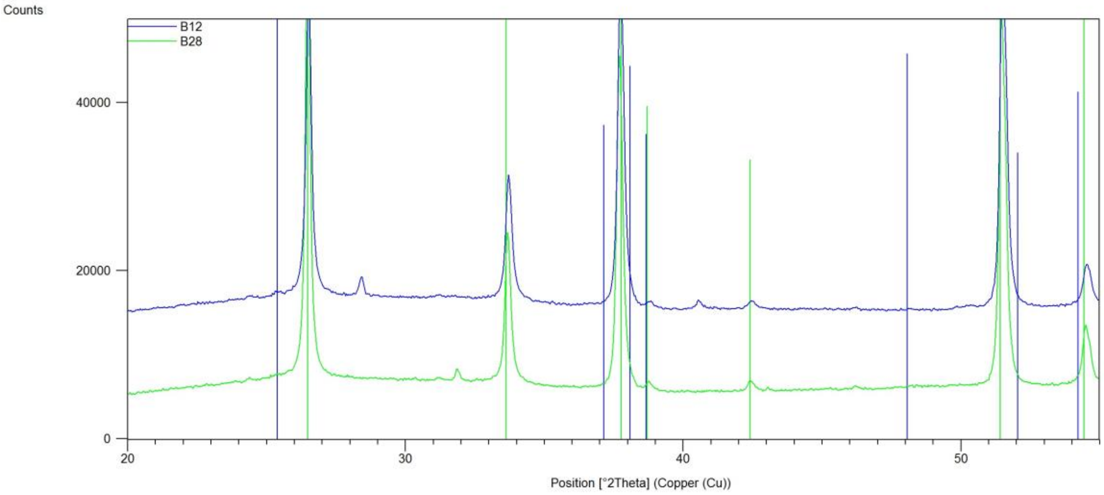


**Figure S1:** XRD patterns of an as-deposited (B28) and an annealed at 500 °C (B12) $TiO_2$ film on an FTO/glass substrate. Precursor 0.2 M TAA, deposition temperature 450 ºC, 70 Spray Cycles. Vertical lines: PDF card no. 04-003-3369 (green) and no. 04-006-1918 (blue) [S1].

***Pattern List:***

| Ref. Code | Compound Name | Mineral Name | Chemical Formula |
|---|---|---|---|
| 04-003-3369 | Tin Oxide | Cassiterite, syn | $SnO_2$ |
| 04-006-1918 | Titanium Oxide | Anatase, syn | $TiO_2$ |

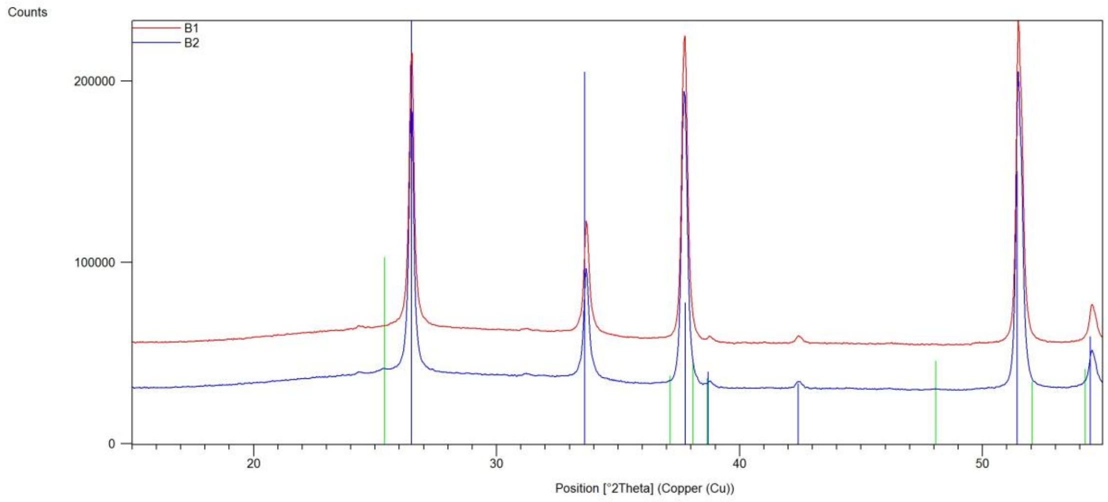


**Figure S2:** XRD patterns of an as-deposited (B1) and an annealed at 500 °C (B2) $TiO_2$ film on an FTO/glass substrate. Precursor 0.2 M TAA- AcAc, deposition temperature 450 ºC, 70 Spray Cycles. Vertical lines: PDF card no. 04-003-3369 (blue) and no. 04-006-1918 (green) [S1].

From the XRD analysis, anatase crystalline structure was not detected for any as-deposited $TiO_2$ films even those consisting of 70 SC (0.2 M TAA and 0.2 M TAA- AcAc), see Fig. S1 (sample B28) and Fig. S2 (sample B1). For post-calcined $TiO_2$ films, anatase can be detected only for films consisting of 70 SC (only one band ($\Theta$ = 25.4°), see Fig. S1. For $TiO_2$ films, consisting of 50 SC and less, anatase can not be detected even after post-calcination.

Similarly, XRD analysis of 70 SC TAA-AcAc film shows that band of anatase ($\Theta$ = 25.4°) is visible only after post-calcination, see Fig. S2, sample B2.

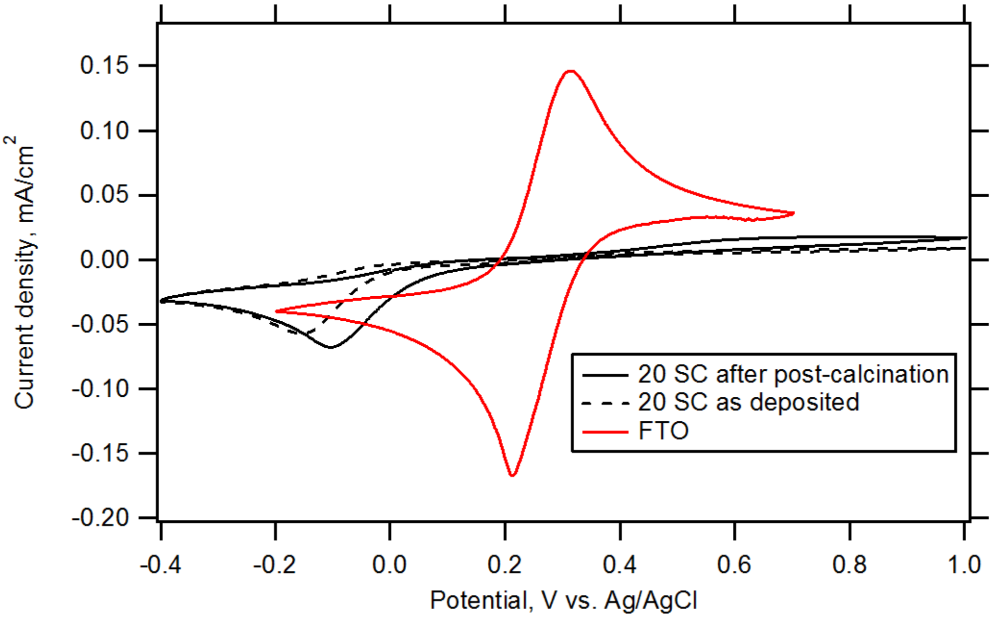


**Figure S3:** Cyclic voltammograms on a bare FTO(B) substrate and that covered by $TiO_2$ films consisting of 20 SC (0.2 M TAA). Scan rate 50 mV/s. Electrolyte solution was 0.5 mM $Fe(CN)_6^{3-/4-}$ in aqueous 0.5 M KCl, pH 2.5.

**Table S1:** Photocurrent densities at a potential of 1.2 V (vs. Ag/AgCl) for calcined $TiO_2$ film prepared from 0.05 M and 0.2 M TAA and from 0.2 M TAA – AcAc.

| Substrate | precursor | No. of SC | j at 1.2 V (vs. Ag/AgCl) [μA $cm^{-2}$] |
|---|---|---|---|
| FTO(A) | 0.05 TAA | 200 | 385 |
| FTO(B) | 0.05 TAA | 200 | 350 |
| FTO(B) | 0.2 TAA | 20 | 390 |
| FTO(B) | 0.2 TAA | 50 | 460 |
| FTO(B) | 0.2 TAA | 70 | 470 |
| FTO(B) | 0.2 TAA-AcAc | 70 | 520 |

# References

[S1] ICDD (**2017**). “Powder Diffraction File,” edited by S.Kabekkodu, International Centre for Diffraction Data, 12 Campus Boulevard, Newton Square, Pennsylvania 19073-3272.
